# Angiosarcoma in a soft tissue sarcoma cohort: real-world patterns and outcomes

**DOI:** 10.3389/fonc.2026.1824033

**Published:** 2026-06-09

**Authors:** Kübra Canaslan, Özge Yetginoğlu, Hasan Oğuz Çetinayak, Emine Burçin Tuna, Tuğba Yavuzşen

**Affiliations:** 1 Department of Medical Oncology, Dokuz Eylül University, İzmir, Türkiye; 2 Department of Radiation Oncology, Dokuz Eylül University, İzmir, Türkiye; 3 Department of Pathology, Dokuz Eylül University, İzmir, Türkiye

**Keywords:** angiosarcoma, real-world outcomes, survival, survival analysis, treatment trajectories

## Abstract

**Introduction:**

Angiosarcoma is a rare, aggressive vascular sarcoma with heterogeneous clinical presentation and limited real-world outcome data.

**Methods:**

We retrospectively reviewed adult soft tissue sarcoma cases managed at Dokuz Eylül University Hospital (January 2019–September 2025) and identified patients with pathologically confirmed angiosarcoma. Clinical, pathological, treatment, and survival data were extracted from electronic medical records and summarized descriptively using Kaplan–Meier methods for time-to-event outcomes.

**Results:**

Among 548 soft tissue sarcoma cases, 12 patients (2.2%) had angiosarcoma; 5 (41.7%) were cutaneous and 7 (58.3%) visceral. Median age was 62 years (range, 19–88), with older age in cutaneous versus visceral disease (median 69 vs 44 years). Metastatic/unresectable disease was present in 9 patients (75.0%) at diagnosis. In the advanced setting (n=9), first-line therapy was paclitaxel-based in 6 (66.7%) and anthracycline-based in 3 (33.3%), achieving disease control in 6/9 (66.7%). Median follow-up was 28.5 months with 7 deaths. Median overall survival (OS) was 9.5 months (95% CI, 6.77–12.22) and median first-line progression-free survival (PFS) was 4.8 months (95% CI, 4.24–5.42).

**Conclusion:**

Angiosarcoma was rare within an institutional sarcoma population but frequently presented at an advanced stage, with poor OS and short PFS despite contemporary therapy. Larger multi-center registries are warranted to refine risk stratification and optimize treatment sequencing.

## Introduction

Angiosarcoma is a rare but highly aggressive vascular sarcoma arising from endothelial cells and accounts for approximately 1–2% of all soft tissue sarcomas (1, 2). Despite its rarity, it poses a substantial clinical burden due to its propensity for rapid local progression, early dissemination, and limited durability of responses to systemic therapy. This aggressive clinical behavior is thought to be driven by substantial biological heterogeneity across angiosarcoma subtypes, with variability in angiogenic pathways, immune milieu, and patterns of dissemination (3).

Population-level data suggest that angiosarcoma incidence is increasing over time. In a large registry-based analysis spanning 2001–2020, the reported incidence reached approximately 3.3 cases per 1,000,000 person-years, and annual case counts roughly doubled between 2001 and 2019 (4). This trend may partly reflect a rising contribution of secondary angiosarcoma. Radiation-associated breast/chest wall tumors after prior cancer treatment are a notable example. More broadly, expanded use of breast-conserving therapy and radiotherapy may increase the pool of patients at risk for radiation-associated disease (4, 5).

Clinically, angiosarcoma encompasses a heterogeneous spectrum of primary sites. Contemporary US data suggest that the majority arise in cutaneous/subcutaneous and breast locations, while a substantial minority present as visceral primaries (6, 7). Stage at presentation is similarly variable. In the same registry analysis, approximately half of patients presented with localized disease, while roughly one quarter had distant metastatic disease at diagnosis, underscoring the frequency of advanced presentation (4, 8).

Management typically integrates local modalities when feasible, including surgery and radiotherapy (1, 9). Systemic therapy remains central for metastatic/unresectable disease. In routine practice, taxane- and anthracycline-based regimens are commonly used in the first-line setting (10, 11) and targeted agents such as pazopanib are frequently employed after progression (12) However, optimal sequencing and expected outcomes vary across cohorts and primary sites, underscoring the need for real-world data. Visceral primaries and metastatic dissemination patterns are not uniform. This further supports granular clinicopathologic characterization and patient-level mapping of treatment courses.

Given the rarity of angiosarcoma and the challenges of prospective evidence generation, real-world single-center cohorts can provide complementary insights. They can contextualize presentation patterns, therapeutic pathways, and survival estimates within routine practice. In this retrospective study, we screened a large institutional soft tissue sarcoma cohort and identified patients with pathologically confirmed angiosarcoma. We aimed to describe demographic and clinicopathological features, compare cutaneous and visceral presentations descriptively, characterize local and systemic treatment patterns (including best response and disease control), and report survival outcomes. Treatment trajectories were additionally summarized using an individual-level swimmer plot.

## Methods

### Study design and patient selection

This was a retrospective, single-center cohort study conducted at Dokuz Eylül University Hospital. We screened all adult patients diagnosed between January 2019 and September 2025 with soft tissue sarcoma (STS). From this source population, we identified patients with a pathologically confirmed diagnosis of angiosarcoma and included those who were ≥18 years of age and had been followed and/or treated at our institution.

Definitive diagnosis required compatible morphology together and supportive endothelial marker expression (e.g., CD31 and/or ERG, with or without CD34/FLI1), as documented in the pathology report. Patients were excluded if the pathologic diagnosis of angiosarcoma was not definitively established, if key clinical dates necessary to ascertain outcomes (date of diagnosis, systemic therapy start, last follow-up/death) were missing, or if follow-up was not available after diagnosis.

### Data collection and variables

Clinical, sociodemographic, and treatment variables were extracted from institutional electronic medical records. Tumor-related data were captured from pathology reports, including primary site, proliferation index when available (Ki-67), and immunohistochemical markers supporting vascular differentiation (e.g., CD31, ERG, CD34, FLI1), as well as other reported markers (e.g., pan-cytokeratin, Human Herpesvirus 8 [HHV8]). Treatment history was recorded by line of therapy, including surgery and resection status (R0/R1 vs non-curative), radiotherapy, and systemic therapy regimens with start dates and dates of progression or treatment discontinuation.

### Outcome definitions

Overall survival (OS) was defined as the time from the date of pathological diagnosis to death from any cause; patients alive at last contact were censored at the date of last follow-up. Progression-free survival (PFS) was defined as the time from the start date of a given systemic treatment line to radiologic and/or clinical progression or death, whichever occurred first; patients without progression were censored at the last disease assessment or last follow-up, as applicable.

Tumor response to systemic therapy was assessed according to RECIST (complete response (CR), partial response (PR), stable disease (SD), progressive disease (PD)) based on available radiologic evaluations and clinical documentation. Best overall response (BOR) was defined as the best response achieved during the corresponding line of therapy, and disease control rate (DCR) as CR + PR + SD among response-evaluable patients.

### Statistical analyses

Given the small cohort size, analyses were primarily descriptive. Continuous variables are reported as median (range) and categorical variables as counts (percentages). Survival outcomes (OS and PFS) were estimated using the Kaplan–Meier method, with median survival times and 95% confidence intervals reported where estimable. No formal between-group hypothesis testing (e.g., log-rank) or multivariable modeling was performed due to the small sample size and limited event counts; subgroup results are presented descriptively. Statistical analyses were performed using SPSS (IBM SPSS Statistics), and figures were generated in R version 4.5 (R Foundation for Statistical Computing).

### Ethics

The study was approved by the Dokuz Eylül University Non-Interventional Research Ethics Committee (decision number 2026/02-22, 12.01.2026). The study was conducted in accordance with the principles of the Declaration of Helsinki. Informed consent was waived due to the retrospective design and use of de-identified data.

## Results

### Patient identification and cohort overview

Between January 2019 and September 2025, 548 patients with STS were screened. Twelve patients (2.2%) had pathologically confirmed angiosarcoma and constituted the study cohort. Primary tumors were classified as cutaneous in 5 patients (41.7%) and visceral in 7 (58.3%).

### Patient and tumor characteristics

The median age at diagnosis was 62 years (range, 19–88). Patients with cutaneous angiosarcoma were older than those with visceral disease (median 69 [53–81] vs 44 [19–86] years). Overall, 5 patients were female (41.7%) and 7 were male (58.3%). Most cases were sporadic/*de novo* (9/12, 75.0%); one case was radiation-associated (8.3%), and two patients (16.7%) had a history of trauma. Given the limited sample size, all subgroup comparisons are descriptive.

### Clinical and pathological features

At diagnosis, 3 patients (25.0%) had localized, resectable and 9 (75.0%) had metastatic/unresectable disease; this distribution was similar across primary site subgroups (**Table 1**). Ki-67 was reported in 4 cases, with a median of 61.2% (range, 15–90). Epithelioid features were documented in one patient (8.3%). HHV8 testing was available in 6 patients and was negative in all (6/6, 100%).

**Table 1:** Demographics and clinicopathological characteristics of patients with angiosarcoma (N=12).

Characteristic	All Patients (N=12)	Cutaneous (N=5)	Visceral (N=7)
Demographics			
**Age at diagnosis**, median (range)	62 (19-88)	69 (53-81)	44 (19-86)
**Sex**, N (%)			
Female	5 (41.7)	2 (40.0)	3 (42.9)
Male	7 (58.3)	3 (60.0)	4 (57.1)
**Etiology**, N (%)			
Sporadic (*De novo*)	9 (75.0)	3 (60.0)	6 (85.7)
Radiation-associated	1 (8.3)	1 (20.0)	0 (0.0)
History of Trauma	2 (16.7)	1 (20.0)	1 (14.3)
Clinical and pathological features			
**Clinical stage**, N (%)			
Localized (Resectable)	3 (25.0)	1 (20.0)	2 (28.6)
Metastatic/Unresectable	9 (75.0)	4 (80.0)	5 (71.4)
**Tumor characteristics**			
Ki-67 Index, median (range) (N=4)	61.2 (15-90)	62.5 (35-90)	52.5 (15-90)
Epithelioid Features, N (%)	1 (8.3)	0 (0.0)	1 (14.3)
HHV8 Status (Negative) (N=6)	6/6 (100)	4/4 (100)	2/2 (100)

HHV8, Human Herpes Virus 8.

Among visceral angiosarcomas (n=7), primary sites were heterogeneous and included the pleura, liver, kidney, thyroid, right atrium, brain, and bone (n=1 each). Among patients presenting with metastatic/unresectable disease (n=9), metastatic involvement commonly included lymph nodes and bone, with additional sites such as skin, abdomen/mediastinal nodal stations, spleen, and bone marrow involvement in one case.

### Treatment patterns

Surgery of the primary site was performed in 8 patients (66.7%), more frequently in cutaneous than visceral angiosarcoma (80.0% vs 57.1%). Curative-intent resection (R0/R1) was achieved in 3 patients (25.0%), while 5 (41.7%) underwent palliative surgery. Among the resected patients (n=3), all received adjuvant radiotherapy (3/3, 100%), and two patients (66.7%) received adjuvant paclitaxel.

Systemic therapy in the advanced setting was administered to 9 patients (**Table 2**). In first-line, paclitaxel-based treatment was most common (6/9, 66.7%) and was used in all cutaneous cases receiving first-line therapy (4/4, 100%), whereas anthracycline-based (AIM) was more frequent in visceral disease (3/5, 60.0%). Best response data showed a DCR of 66.7% (6/9), including 80.0% (4/5) of visceral and 50.0% (2/4) in cutaneous cases; progressive disease as best response occurred in 3/9 patients (33.3%).

**Table 2:** Treatment patterns and outcomes of patients with angiosarcoma (n=12).

Characteristic	All Patients (N=12)	Cutaneous (n=5)	Visceral (n=7)
Primary treatment			
**Surgery of Primary Site**, N (%)	8 (66.7)	4 (80.0)	4 (57.1)
Curative Intent (R0/R1)	3 (25.0)	1 (20.0)	2 (28.6)
Palliative	5 (41.7)	3 (60.0)	2 (28.6)
**Adjuvant Therapy** (for resected)	**(N=3)**	**(N=1)**	**(N=2)**
Radiotherapy	3 (100)	1 (100)	2 (100)
Chemotherapy (Paclitaxel)	2 (66.7)	0 (0.0)	2 (100)
Systemic Therapy (Advanced Setting)	(N=9)	(N=4)	(N=5)
**First-line regimen**, N (%)			
Taxane-based (Paclitaxel)	6 (66.7)	4 (100)	2 (40.0)
Anthracycline-based (AIM)	3 (33.3)	0 (0.0)	3 (60.0)
**Best response to 1st line**, N (%)			
Disease Control (CR+PR+SD)	6 (66.7)	2 (50.0)	4 (80.0)
Progressive Disease	3 (33.3)	2 (50.0)	1 (20.0)
**Second-line Therapy**, N (%)	**(N=5)**	**(N=2)**	**(N=3)**
Pazopanib	4 (80.0)	1 (50.0)	3 (100)
Doxorubicin	1 (20.0)	1 (50.0)	0 (0.0)
**Third-line Therapy**, N (%)	**(N=3)**	**(N=1)**	**(N=2)**
Liposomal Doxorubicin	2 (66.7)	1 (100)	1 (50.0)
Regorafenib	1 (33.3)	0 (0.0)	1 (50.0)

AIM, Doxorubicin- ifosfamide – mesna; CR, Complete Response; PR, partial response; SD, stable disease. Bold numerals indicate the number of patients in the relevant subgroup.

**Figure 1:**
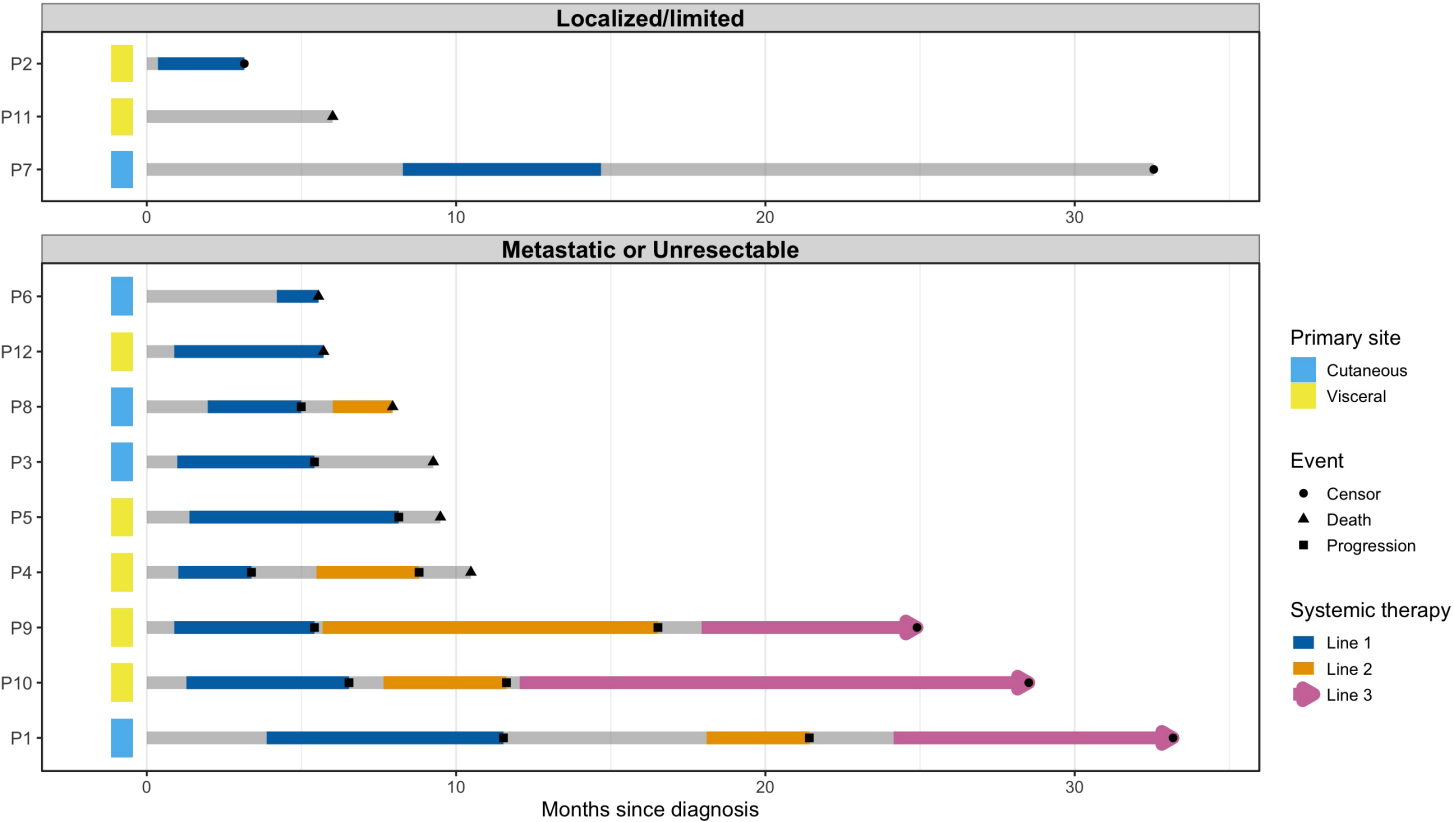
Swimmer plot of individual treatment courses in angiosarcoma. Each horizontal lane represents one patient, with time shown in months from diagnosis. The light-gray bar indicates the observation period from diagnosis to last follow-up or death. Colored segments denote exposure to systemic therapy by line of treatment (Line 1, Line 2, and Line 3), with arrowheads indicating ongoing treatment at the time of last follow-up. Patients are displayed in two panels according to extent of disease at diagnosis (Localized/limited vs Metastatic/unresectable). The narrow-colored strip on the left indicates primary tumor site (cutaneous vs visceral). Symbols mark key events: squares indicate progression at the end of a treatment line, circles indicate censoring (alive at last follow-up), and triangles indicate death.

Second-line systemic therapy was administered in 5 patients, most commonly pazopanib (4/5, 80.0%), with doxorubicin used in one patient (20.0%). Third-line therapy was given to 3 patients, including liposomal doxorubicin in two (66.7%) and regorafenib in one (33.3%) (**Table 2**). Individual treatment trajectories are summarized in **Figure 1**.

### Survival outcomes

The median follow-up was 28.5 months, during which 7 deaths occurred. In the overall cohort, the median OS was 9.5 months (95% CI, 6.77–12.22), with a mean OS of 17.0 months (95% CI, 9.74–24.29). Median OS was 9.5 months among patients with metastatic/unresectable disease (95% CI, 8.82–10.17), whereas the median OS for the limited/resectable group was not estimable due to limited events/censoring. When stratified by primary site, median OS was 9.27 months in cutaneous angiosarcoma (95% CI, 6.44–12.09) and 9.50 months in visceral angiosarcoma (95% CI, 4.13–14.86); these subgroup estimates are presented descriptively given the small sample size.

For first-line PFS, the median was 4.83 months (95% CI, 4.24–5.42). When stratified by primary site, median first-line PFS was 4.44 months in cutaneous angiosarcoma (95% CI, 1.40–7.47) and 4.83 months in visceral angiosarcoma (95% CI, 4.21–5.45). Given the small sample size, particularly in the localized/resectable subgroup, subgroup estimates are presented descriptively with 95% CI and no formal between-group hypothesis testing was performed.

## Discussion

In this single-center cohort study, angiosarcoma represented a small fraction of all soft tissue sarcomas (2.2%), with the majority of patients (75.0%) presenting with advanced or unresectable disease. Despite the implementation of contemporary therapeutic regimens, survival outcomes remained poor, characterized by a median OS of 9.5 months and a median first-line PFS of 4.8 months. These findings are consistent with the aggressive clinical course and limited durability of treatment responses.

The median OS of 9.5 months observed in our cohort underscores the aggressive nature of this malignancy and aligns with results from other real-world datasets. Survival in angiosarcoma is heavily dependent on stage and primary site; while localized disease may allow for multi-year survival (13, 14), outcomes for metastatic/unresectable disease frequently remain under one year (15–18). Metastatic disease at diagnosis and liver primary site have been associated with inferior outcomes (6, 19). Notably, the median OS for the limited/resectable group was not estimable due to censoring, highlighting the critical role of early surgical intervention and adjuvant radiotherapy in extending survival.

The anatomic distribution in our series differed from large population-based data. While earlier cohorts typically reported a predominance of cutaneous angiosarcoma (4, 20), visceral primaries slightly predominated in our series, likely reflecting tertiary-center referral and case mix differences. Importantly, our data also demonstrated marked anatomic heterogeneity among visceral primaries. Visceral tumors comprised a slight majority (58.3%) and involved multiple organ sites (pleura, liver, kidney, thyroid, right atrium, brain, and bone), supporting the view that visceral angiosarcoma represents a spectrum rather than a single entity. Population-based datasets from the US and Denmark similarly demonstrate that visceral angiosarcoma can arise in virtually any organ, with the liver among the most frequently involved sites, supporting the concept of substantial anatomic heterogeneity (21, 22).

Metastatic spread in angiosarcoma is heterogeneous across cohorts; however, the lung is consistently reported as a common metastatic site, with variable involvement of bone, liver, and brain (6, 23). In our metastatic/unresectable cases, lymph node and bone involvement were frequent, with additional sites including skin, abdominal/mediastinal nodal stations, spleen, and bone marrow, supporting the need for careful baseline staging and frequent reassessment in routine practice. Clinically, while sarcomas are traditionally considered to disseminate predominantly via hematogenous spread (24), the frequency of lymph node involvement in our cohort suggests that angiosarcoma may also exhibit a more carcinoma-like dissemination pattern in selected patients. This supports comprehensive baseline staging, particularly of the thorax and bones, and low threshold repeat imaging during early treatment.

We observed patients with visceral disease were considerably younger than those with cutaneous presentations (median 44 vs. 69 years). While cutaneous cases often arise in the context of UV damage or chronic lymphedema in older populations (25, 26), the observed age-related divergence between primary sites in our cohort is clinically relevant; however, the small sample size and lack of molecular profiling preclude definitive biological inferences, underscoring the need for confirmation in larger, molecularly annotated studies.

Primary and secondary angiosarcoma may differ in outcomes; however, reported survival differences are often confounded by baseline stage distribution (18). In our cohort, only one patient had a clearly documented predisposing factor (prior radiotherapy), while the remaining cases lacked an identifiable risk factor in the available retrospective records, limiting etiologic subgrouping and cross-cohort comparisons.

Local modalities remain foundational in localized/resectable disease and our cohort reflects this principle. Surgery was performed in 66.7% of our cohort, although only a minority achieved curative-intent margins (R0/R1) (25%) while the remainder underwent palliative surgery/metastasectomy, illustrating that operability does not necessarily translate into curability in angiosarcoma. All resected patients received adjuvant radiotherapy, consistent with an institutional strategy to mitigate the high risk of local relapse. Retrospective series in localized cutaneous head and neck angiosarcoma similarly support multimodal local approaches, including radiotherapy, to improve local control and potentially survival (9). Even with negative margins, subclinical microscopic spread can be substantial, and adjuvant radiotherapy is commonly recommended for high-risk features such as large tumors (≥5 cm), head and neck/scalp location, multifocality, and close or positive margins. Adjuvant treatment is typically delivered to the operative bed and at-risk tissues, with total doses around 60 Gy for margin-negative resections and 66–70 Gy for margin-positive disease, often using conformal techniques (e.g., Intensity-Modulated Radiation Therapy/Volumetric Modulated Arc Therapy) to optimize target coverage in dermal involvement (1, 27). Although radiotherapy can improve local control, OS is frequently constrained by distant relapse (28), underscoring the need for effective systemic strategies within a multidisciplinary approach.

Systemic treatment was administered to most patients in the advanced setting (n=9). First-line paclitaxel-based regimens predominated overall (66.7%) and were used in all cutaneous cases receiving systemic therapy, whereas anthracycline-based therapy was more common in visceral disease. This likely reflects pragmatic real-world selection, where weekly paclitaxel is favored for feasibility and tolerability in older patients while anthracycline-based therapy may be reserved for selected fit patients with visceral disease and higher disease burden.

In our cohort, first-line disease control was achieved in two-thirds of treated patients (DCR 66.7%), yet benefit was short-lived (median first-line PFS 4.8 months), suggesting that initial chemosensitivity may not translate into durable control. This pattern is consistent with prospective and real-world paclitaxel experiences, including ANGIOTAX and European Organisation for Research and Treatment of Cancer (EORTC) Soft Tissue and Bone Sarcoma Group trials, in which time to progression approximated 4 months and median OS remained limited (29, 30). Real-world cohorts have reported similar PFS/OS estimates with weekly paclitaxel, and adverse prognostic features such as liver involvement may further influence outcomes (31).

Later-line treatment patterns also mirrored routine practice. Pazopanib was the most frequently used second-line agent (4/5; 80%), followed by liposomal doxorubicin and regorafenib in a small number of patients. Although our sample precludes efficacy comparisons, documenting sequencing remains clinically relevant because treatment decisions in angiosarcoma are often made under substantial uncertainty, and reported activity of later-line agents is generally modest (32, 33).

This study has limitations inherent to its retrospective, single-center design and small sample size, which restrict subgroup comparisons. Pathology reporting and ancillary testing reflected routine clinical practice and therefore showed some variability, with selected immunohistochemical markers such as Ki-67 and HHV8 not assessed in all patients. Treatment selection and sequencing were non-random and influenced by clinical factors, so findings should be interpreted descriptively rather than causally.

Strengths include a large institutional STS source population enabling estimation of angiosarcoma frequency, detailed line-by-line treatment annotation, and patient-level visualization of trajectories with a swimmer plot. This series also contributes real-world data from Türkiye and highlights the diversity of visceral primary sites. Larger multi-center registries are needed to validate these observations and optimize risk stratification and treatment sequencing.

## Conclusion

In this single-center angiosarcoma cohort, most patients presented with advanced disease and outcomes remained poor despite contemporary therapy. Although disease control was frequently achieved, benefit was short-lived, underscoring the need for more durable systemic strategies, especially for metastatic/unresectable presentations. Patient-level trajectory mapping highlighted the heterogeneity of visceral primaries and treatment courses, supporting the development of larger multi-center registries to refine risk stratification and optimize sequencing.

## Data Availability

The datasets presented in this article are not readily available because they contain de-identified patient data and are subject to institutional privacy and ethics regulations. Requests to access the datasets should be directed to the corresponding author.
